# Test-enhanced learning in Neuroanesthesia for the First Year anesthetic residents: a randomized controlled trial

**DOI:** 10.1186/s12909-024-05887-0

**Published:** 2024-08-23

**Authors:** Manee Raksakietisak, Vasu Lertsiripatarajit, Naiyana Aroonpruksakul, Narin Plailaharn, Kasana Raksamani

**Affiliations:** 1 Department of Anesthesiology, Faculty of Medicine Siriraj Hospital, Mahidol University, 2 Wanglang Road, Bangok Noi, Bangkok, 10700 Thailand; 2 Department of Anesthesiology, Faculty of Medicine, Khon Kaen University, Khon Kaen, Thailand

**Keywords:** Test enhancing learning, Anesthesiology, Residents, Neuro-anesthesia

## Abstract

**Background:**

Test enhancing learning (TEL) had shown a significant effect in promoting the learning of many learning contents. However, its effect on the postgraduate medical level was unclear. This study aimed to investigate the effect of TEL in 1st year anesthesiology residents learning neuroanesthesia.

**Method:**

The residents were randomized to either group A, which was assigned to do the intervention exam (exam A) for two times during learning in neuroanesthesia, or group B, which studied in the same environment without doing the exam. All participants were assigned to do the assessment exam (exam B) at one month after the end of the rotation. All of the exams were ten multiple choice questions (MCQ). Since the anesthesia residents rotated to neuroanesthesia for two weeks twice during the first year, we conducted the experiments twice, using exams that covered both basic science (BS) and clinical science (CS) topics.

**Results:**

There was no significant difference in mean ± SD of the scores for assessment exams asking about the basic science topic (BS_B) [group A (5.25 ± 2.05) VS group B (4.90 ± 1.80); *p* = 0.570] and the clinical science topic (CS_B) [group A (6.30 ± 1.26) VS group B (5.95 ± 1.61); *p* = 0.448].

**Conclusion:**

This study showed null findings on the effect of TEL on learning in residents of the first year of anesthesiology. More studies on TEL were required to confirm the effect of TEL and find the appropriate test format that could enhance learning for post-graduate medical trainees.

## Introduction

Anesthesia residents are required to master a vast amount of knowledge in a competency-based education system during their postgraduate training [1]. Competency-based medical education (CBME) emphasizes the achievement of specific skills and competencies, focusing on the residents’ ability to perform tasks to a predetermined standard [2]. This approach ensures that residents not only acquire theoretical knowledge but also develop practical skills and professional behaviors essential for patient care [3]. Retaining this knowledge can be challenging due to the demands of busy clinical work, high-pressure situations, and learning in complex environments [4]. Senior anesthesia residents have reported facing more challenges with academic education than with clinical learning experiences [5]. The implementation of entrustable professional activities (EPA) has been proven to bridge the gap between desired competency and clinical practices [6]. EPAs provide a structured framework that allows for the progressive entrustment of clinical responsibilities based on demonstrated competencies, enhancing the transition from learning to practice [6, 7]. However, assessments focusing mainly on knowledge are still necessary to enhance other competencies [8].

The concept of test-enhanced learning (TEL) has been shown to improve memory and retrieval processes to learn new information [9, 10]. Testing requires learners to actively retrieve data from memory, which strengthens retrieval pathways, making retrieval of these data easier in the future [11, 12]. It also improves future learning by increasing correct recall [13, 14]. Studies in cognitive psychology have shown that TEL encourages focusing attention on content, promotes task-relevant behaviors such as taking notes, and reduces overall cognitive demand [15]. TEL has demonstrated its effectiveness in various age groups, from elementary-aged students to middle-aged and older adults [16–18]. It has been applied at many educational levels, including medical education, and has shown significant effects in improving learning [9, 11, 15, 19–23]. However, most TEL studies have been conducted with learners developing foundational knowledge and have produced inconsistent results at the postgraduate medical level [22].

In the context of residency training, particularly within a CBME framework, TEL can play a crucial role [1, 2]. Residency training is characterized by its demanding nature, where residents must balance clinical duties with continuous learning and skill development [2, 5]. TEL can help residents retain critical information and integrate it into their clinical practice, addressing one of the main challenges of postgraduate medical education—the retention and retrieval of extensive complex information [1, 2]. This study aimed to investigate the effect of TEL on 1st-year anesthesiology residents’ learning of neuroanesthesia. We used multiple choice questions (MCQ) as the intervention, comparing them with residents who followed the standard training program. Furthermore, this study sought to identify other factors that could improve the learning of neuroanesthesia in first-year residents.

## Methods

### Study context

The residency training program in anesthesiology spans three years, employing a competency-based approach and an assessment system known as Entrustable Professional Activities (EPA). The clinical clerkship for neuroanesthesia includes rotations in neurosurgical operating theaters twice for two-week durations in the first year, one month in neuroradiology suites in the second year, and one month in neurosurgical operating theaters in the third year.

Learning experiences are enriched through one-on-one clinical supervision, simulation, prerecorded online lectures, exam pools, and self-directed learning, all integrated within the EPA system. During the neuroanesthesia clerkship, all residents were supervised by attending neuroanesthesiologists, with the level of supervision determined by the level of entrustment assessed by the EPA. In their second year, each resident participated in simulation sessions focused on neuroanesthesia for decision-making and anesthesia management. Additionally, at all times, residents had access to prerecorded online lectures on neuroanesthesia via the curriculum’s e-learning platform. At the conclusion of the first year, all first-year residents undertake a final exam in neuroanesthesia, consisting of 20 questions that encompass both basic and clinical sciences. This end-of-year exam is an integral part of the training evaluation.

### Methods

This study received approval from the Siriraj Institutional Review Board (Si 642/2020). Participants were recruited from the first-year residents of the Department of Anesthesiology, Faculty of Medicine, Siriraj Hospital, Mahidol University, during the academic year 2020–2021. The written informed consent was obtained from all participants before the data collection. However, 1st-year residents who began their rotation in neuro-anesthesia in the first month were excluded, as they were new trainees requiring a period for learning adaptation.

Participants were randomly divided into two groups: Group A and Group B, using block randomization. Group A, which served as the study group, underwent a pre-test, post-test, and final test. In contrast, Group B only took the final test. On the first day of their neuroanesthesia rotation (D1), only the participants in Group A took the pre-test (BS_A_1) and then repeated the same test on the last day (D15) of the rotation, termed the post-test (BS_A_2). Group A participants were instructed not to share the questions with their peers. After one month, at the end of the rotation (D45), both groups took the final test (BS_B).

**Fig. 1:**
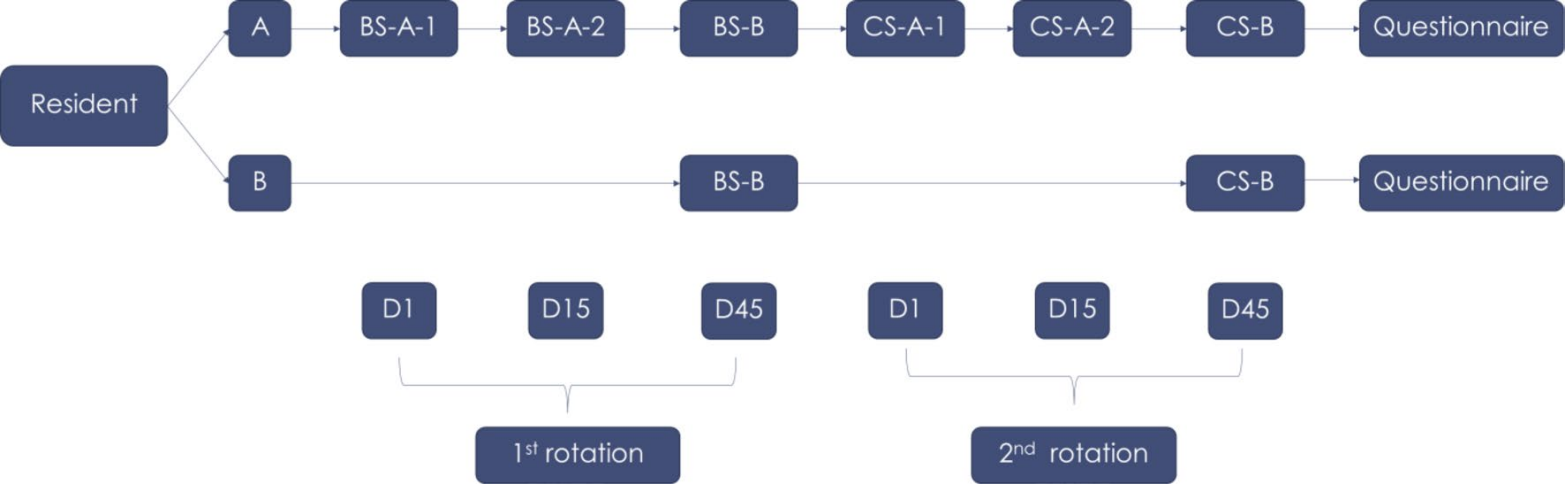
Sequence of tests for participants in each group- BS_A_1 – the first attempt for the basic science exam A, BS_A_2 – the second attempt for the basic science exam A, CS_A_1– the first attempt for the clinical science exam A, CS_A_2 – the second attempt for the clinical science exam A, BS_B – the attempt for the basic science B, CS_B – the attempt for the clinical science B – D1 – 1^st^ day of rotation, D15 – 15^th^ day of rotation (last day), D45 – 45^th^ day (one month after the end of rotation)

The same process was repeated during the second rotation of neuroanesthesia, this time using the test for the clinical science topic. After completing all the tests, participants were asked to fill out questionnaires designed to gather demographic data, opinions about test-taking, learning habits, and the most effective methods for learning neuroanesthesia. The questionnaire was developed by a team of researchers by reviewing relevant literature and aligning it with the study’s objectives. It was a paper-based questionnaire administered to ensure ease of access and completion. Participation in the questionnaire was voluntary and anonymous, ensuring that responses could not be traced back to individual participants. The research methods are illustrated in Fig. 1.

In this study, a total of four sets of multiple-choice questions (MCQ) were used. These comprised two sets focused on the basic science of neuro-anesthesia (BS topic) - specifically, Basic Science Exam A (BS_A) and Basic Science Exam B (BS_B) - and two sets centered on the clinical science of neuro-anesthesia (CS topic): Clinical Science Exams A (CS_A) and B (CS_B).

Both BS_A and BS_B contained ten questions that covered the same content but with varied questions. The same structure was observed for CS_A and CS_B. The decision to include ten questions in each exam was based on a previous study, which indicated that ten questions struck the right balance of efficiency and effectiveness [13]. Each question in these exams provided four answer choices. The “A” exams (BS_A and CS_A) functioned as intervention exams, while the “B” exams (BS_B and CS_B) served as assessment exams (refer to Fig. 1).

All questions were selected from an existing exam bank or specifically created for this study, aligning with the curriculum test blueprint. Three neuroanesthesiologists, each with over 5 years of experience in the field, evaluated the validity and suitability of every question.

- D1–1st day of rotation, D15–15th day of rotation (last day), D45- 45th day (one month after the end of rotation).

### Statistical analysis

The sample size for this study was determined on the basis of a previous study. In that study, the average ± SD scores for the TEL group and the self-learning group were 53.91 ± 1.72 and 48.0 ± 1.8, respectively, representing a mean difference of 5 points out of 100 possible [16]. Given that the full score of our tests was 10, we projected the mean difference to be 1 point. After calculating the effect size and consulting with statisticians, it was determined that 34 participants, split equally into two groups, would be needed to achieve an 80% power. To compensate for possible participant withdrawals or for those who might not complete all tests according to the protocol, we decided to include an additional 10%, bringing the total to approximately 38 participants.

Given that the annual intake of first-year residents in the Department of Anesthesiology, Faculty of Medicine, Siriraj Hospital, Mahidol University ranges from 27 to 29, it was necessary to attract participants from at least two academic years.

Descriptive statistics were employed to assess demographic data, such as gender, and to capture participants’ perceptions about their learning. Group comparisons were made using Chi-square tests and Fisher’s exact tests. The Student’s t test was used to compare the scores of individual tests and the end-of-year exams between the two groups. A p-value below 0.05 was deemed statistically significant. Data analysis was carried out using IBM SPSS for Windows, version 23.0.

## Results

**Fig. 2:**
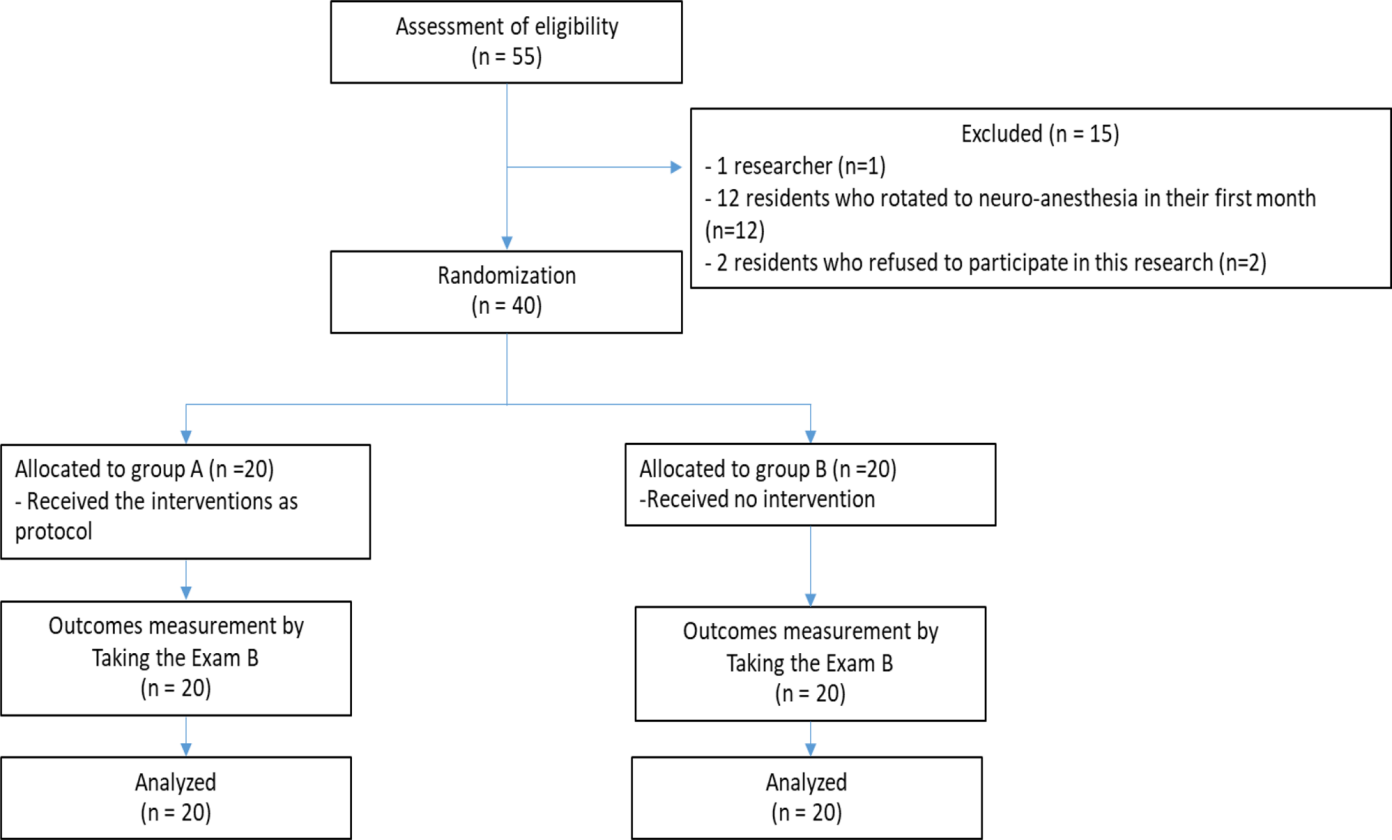
CONSORT flow

### Demographic data

This study was conducted with every first year resident who consented to participate. Fifteen residents were excluded before randomization. A total of 40 participants completed all the exams according to the research protocol, with 20 participants in each group, as depicted in Fig. 2. There were no statistically significant differences in terms of gender, age, grade point average (GPA), number of internship years, type of post-training hospital workplace, or availability of neuroanesthesia service at post-training hospital workplaces between the groups, as detailed in Table 1.

**Table 1:** Demographic data of the participants

	Group A (*N* = 20)	Group B (*N* = 20)	*p*-value
Gender			0.235
- Female: male	14: 6	18: 2	
Age (years)	27.75 ± 0.79	27.8 ± 0.95	0.652
Grade Point Average (GPA)	3.56 ± 0.25	3.47 ± 0.22	0.242
Number of years of internship (years)			0.540
- 2	5 (25)	6 (30)	
- 3	15 (75)	13 (65)	
- 4	0 (0)	1 (5)	
Type of post training hospital workplace			0.620
- Medical school hospital	1 (5)	2 (10)	
- Regional hospital	2 (10)	3 (15)	
- Large general hospital	4 (20)	3 (15)	
- Small general hospital	4 (20)	7 (35)	
- Unknown	9 (45)	5 (35)	
Available of neuro-anesthesia service of post training hospital work place			0.209
- yes	7 (35)	10 (50)	
- no	5 (25)	7 (35)	
- unknown	8 (40)	3 (15)	

Data presented as a number (%) or mean ± SD as appropriate

### Effect of TEL

The score for the first assessment exam, BS_B, did not differ significantly between Group A (5.25 ± 2.05) and Group B (4.90 ± 1.80) (*p* = 0.570). Similarly, the score for the second assessment exam, CS_B, for Group A (6.30 ± 1.26) and Group B (5.95 ± 1.61) also did not show a significant difference (*p* = 0.448). Furthermore, there were no significant differences between the end-of-year exam scores of the participants in Group A (13.7 ± 1.72) and Group B (13.1 ± 2.05) (*p* = 0.322), as illustrated in Table 2.

**Table 2:** Score of the basic science B and clinical science B test and midterm exam score

	Group A (*N* = 20)	Group B (*N* = 20)	*p*-value
Intervention test			
- BS_A_1 score	4.35 ± 1.87	N/A	
- BS_A_2 score	5.75 ± 1.29	N/A	
- CS_A_1 score	7.05 ± 1.36	N/A	
- CS_A_2 score	7.20 ± 1.15	N/A	
Assessment test			
- BS_B score	5.25 ± 2.05	4.90 ± 1.80	0.570
- CS_B score	6.30 ± 1.26	5.95 ± 1.61	0.448
End of the year exam score	13.7 ± 1.72	13.1 ± 2.05	0.322

Data presented as mean ± SD

BS_A_1 score – the first attempt scores for basic science exam A, BS_A_2 score – the second attempt scores for basic science exam A, CS_A_1 score – the first attempt scores for clinical science exam A, CS_A_2 score – the second attempt scores for clinical science exam A, BS_B score – the score for basic science B score, CS_B score – the score for clinical science B score

The full score of the intervention and assessment test was 10, the full score of the end-of-year exam was 20

### Factors enhancing learning in Neuroanesthesia

Based on the questionnaires administered at the research’s conclusion, 65% of participants in group A reported that they felt they benefited greatly and very much from taking the MCQ exam compared to 50% of group B. The optimal parameters for the exam, as indicated by the participants, were 15 questions with a duration of 20 min, to be taken twice. After completing the exams, the majority of the participants reported spending 0–2 h on additional reading on neuro-anesthesia, as shown in Table 3.

**Table 3:** MCQ questionnaires

	Group A (*N* = 20)	Group B (*N* = 20)	*p*-value
Learning from doing MCQ			0.171
- Very much	3 (15)	0	
- Much	10 (50)	10 (50)	
- Fair	7 (35)	10 (50)	
- Little	0	0	
- Very little	0	0	
Appropriate number of questions for each test			0.278
- 5	0	0	
- 10	11 (55)	6 (30)	
- 15	7 (35)	11 (55)	
- > 15	2 (10)	3 (15)	
Appropriate duration to take the 10 questions MCQ test.			0.506
- 10 min	2 (10)	1 (5)	
- 15 min	10 (10)	7 (35)	
- 20 min	8 (40)	11 (55)	
- Unlimited	0 (0)	1 (5)	
Appropriate times to perform test			0.386
- 0	1 (5)	1 (5)	
- 1	3 (15)	6 (30)	
- 2	14 (70)	13 (65)	
- More than 2	2 (10)	0 (0)	
Times spent on self-learning after doing the test			0.399
- 0	2 (10)	5 (25)	
- 0–2 h	14 (70)	10 (50)	
- 2–5 h	4 (20)	4 (20)	
- More than 5 h	0 (0)	1 (5)	

Data presented as number (%)

Regarding which methods most improved their learning in neuroanesthesia, some methods consistently ranked highly. To analyze the results, we assign scores of 3, 2, and 1 to the methods voted as the first, second, and third most learning-enhancing methods, respectively. The discussion of cases with supervisors for crucial insights received the highest score, followed by providing anesthesia for patients under close supervision by supervisors, and then providing anesthesia with distance supervision. These results are presented in Table 4.

**Table 4:** Learning-enhancing methods in participants opinions

	The most learning-enhancing methods			
	First (*N* = 40)	Second (*N* = 40)	Third (*N* = 40)	Score*
Preoperative discussion with supervisors	5	3	3	24
Providing anesthesia for patients with close supervision	6	12	9	51
The entrustable professionalism activities (EPA) evaluation	2	0	7	13
Discussing the case with supervisors for important points	20	12	1	85
Self-learning	3	6	6	27
Self-testing	0	1	5	7
Providing anesthesia with distance supervision	4	6	9	33

Score calculated by giving a score of 3, 2 and 1 for each method that was voted as first, second, and third most learning-enhancing methods for each participant, respectively

## Discussion

This study is a randomized controlled trial that investigated the effect of test-enhanced learning (TEL) on the knowledge of neuroanesthesia of first year anesthesiology residents. Participants who took the intervention MCQ tests during their neuroanesthesia rotation did not show a significantly different score on either of the assessment exams compared to participants who did not take the intervention exam. However, participants in the study group reported that the MCQ test had an educational impact, as they felt they learned from taking the test.

This result suggests that TEL may not have had a significant effect on the residents’ learning, unlike previous studies that reported a significant effect of TEL [16, 22]. Therefore, we examined the differences between our protocol and theirs. One study used short answer questions (SAQ) and provided answers for each question as feedback on learning quizzes [22]. Consequently, we explored the reported effects of these two factors: the test format and the provision of feedback on TEL. The primary factor was the test format. Tests that encouraged students to generate answers on their own might offer a more potent TEL effect [15, 24]. From a previous review, it was reported that there is an advantage of SAQ over MCQ and a benefit of context-rich MCQs (which require the application of knowledge) over context-free MCQs [20]. However, different test formats may be more suitable for various learning content and outcomes. MCQ tests might offer greater benefits for memorization and fact retention, while SAQ tests might be more useful for conceptual and abstract learning content [19]. Notably, the effects of TEL are stronger when the formats of the intervention and assessment tests are identical. It is essential to determine whether the increased scores result from improved knowledge or merely from familiarity and recognition [19, 20]. Another factor to consider is providing feedback after the test. In this study, we did not show participants their scores or provide feedback on their responses to the intervention tests. The significance of feedback lies in its ability to correct erroneous responses during the test and to prevent students from being exposed to inaccurate information [24]. Feedback is also seen as an indirect testing effect since it guides students to focus on content areas that might require more attention [25, 26]. The TEL effect is marginally better when feedback is provided after the test [19]. Interestingly, from the questionnaires given to the participants at the end of the protocol, some expressed a desire to know the correct answers.

The results of the questionnaires, which focus on self-reported factors that improve learning, showed that discussing cases with supervisors about vital points obtained the highest score. Participants noted that administering anesthesia under both close and distant supervision augmented their learning in neuroanesthesia. In contrast, self-testing ranked lowest. This pattern is consistent with the complexity of the competency-based curriculum in residency training, indicating that clinical content cannot be fully augmented by knowledge tests alone [4]. Integration of the cognitive, psychomotor, and affective domains of learning and assessment can be maximized using a competency-based approach [1]. The learning format in neuroanesthesia not only demands content knowledge but also an in-depth understanding of crucial operational points - insights frequently gleaned from hands-on experience. Consequently, introducing TEL to this kind of learning context might require modifications. The participants also highlighted the educational impact of the MCQ testing in the intervention group of the questionnaires. However, studies have illustrated that for assessments to offer educational advantages, they should not only cater to content but also to the depth and retention of learning [27]. A pivotal factor is the assessment’s ability to inspire learning motivation, be it through score acquisition, feedback, or interactions with assessors [28, 29]. In our approach using the MCQ examination, learning motivation might stem from the exam’s familiar format and the feedback participants received in the form of scores.

We acknowledge several limitations in this study. First, the research was conducted in two academic cohorts, which could influence various contexts, such as the learning environment and students’ perceptions and motivations [30, 31]. Second, the study was conducted in a single learning center. This environment may differ from others and is a crucial factor in learning. Third, the study sample size might have been too small to reveal a significant effect of TEL. Additionally, there was a lack of control between the groups, which could introduce variability and affect the results. Lastly, the limitations of the test questions included in the MCQ test should be considered. The selection process, while rigorous, may still have resulted in questions that did not fully encompass all relevant aspects of neuroanesthesia, potentially impacting the assessment’s comprehensiveness.

Although we did not find a significant effect of TEL on learning neuroanesthesia in this study, participants reported an educational impact from taking MCQs. Further research is needed to confirm TEL’s effect on learning and to design test formats that can effectively enhance learning.

## Data Availability

The datasets used and/or analysed during the current study are available from the corresponding author on reasonable request.

## Declarations

### Clinical registration number

The clinical trial registration is not applicable due to the nature of the research, which does not involve patient care.
